# Supplementary material for: Alteration of Neural Network and Hippocampal Slice Activation through Exosomes Derived from 5XFAD Nasal Lavage Fluid
Source: Int J Mol Sci. 2023 Sep 14;24(18):14064. doi: 10.3390/ijms241814064 (PMC10531257; doi:10.3390/ijms241814064)
Supplement: Supplementary file 1 [file ijms-24-14064-s001.zip › ijms-2576747-Figures and Movies caption.pdf]

Supplementary Figure S1. A. Experimental flow of functional tests of exosome in the primary cortical culture and the organotypic slice culture with the successive HD MEA recording. The red box in the picture of the HD MEA system represents the recording area of the CMOS chip with the perfusion ports and reference electrode. After the experiment, data analysis is conducted first for the topological properties of samples and then for further advanced analysis like the connectivity map characterization. B. Schematic configuration of CMOS chip with its physical specifications.

Supplementary Figure S2. A. a. Nissl staining image of brain slice after cortex sectioning in a control animal. (scale bar = 500 μm) b. Nissl staining image from the cortical area in the box region of A-a. (scale bar = 100 μm) B. a. Neuronal culture image under an uplight microscope cultured on CMOS MEA chip. (scale bar = 100 μm) b-d. Organotypic hippocampal slices cultured on the translucent mesh membranes. The membrane sides are facing upward while the tissue surface contacts the CMOS MEA chip. Control (b), Aβ42 (c), and 5XFAD (d) slices applied in Figure 4. (scale bar = 500 μm)

Supplementary Figure S3. (A) Distribution histogram of node probability to average path length in number. Blue for DIV 7, green for DIV 10, and red for DIV 13. (B) Distribution histogram of node probability to node degree in a number of links. Blue for DIV 7, green for DIV 10, and red for DIV 13. Cumulative graphs for the node counts are overlaid in the histogram graphs.

Supplementary Figure S4. (A) CSD waveform amplitude over time separated into sources (red) and sinks (blue) in control OHSC. (B) CSD waveform amplitude over time separated into sources (red) and sinks (blue) in Aβ42 OHSC. (C) CSD waveform amplitude over time separated into sources (red) and sinks (blue) in 5XFAD NLF exosome-treated OHSC.

Supplementary Figure S5. High resolution image of Figure 3A.

Movies S1–S3. CSD amplitude changes over time in 3D graph and geographical location of control (Movie S1), Aβ42 (Movie S2), and 5XFAD NLF exosome treated OHSC (Movie S3). Red for sources and blue for sinks.
