# Supplementary material for: Alteration of Neural Network and Hippocampal Slice Activation through Exosomes Derived from 5XFAD Nasal Lavage Fluid
Source: Int J Mol Sci. 2023 Sep 14;24(18):14064. doi: 10.3390/ijms241814064 (PMC10531257; doi:10.3390/ijms241814064)
Supplement: Supplementary file 1 [file ijms-24-14064-s001.zip › ijms-2576747-supplementary.pptx]

## Slide 1
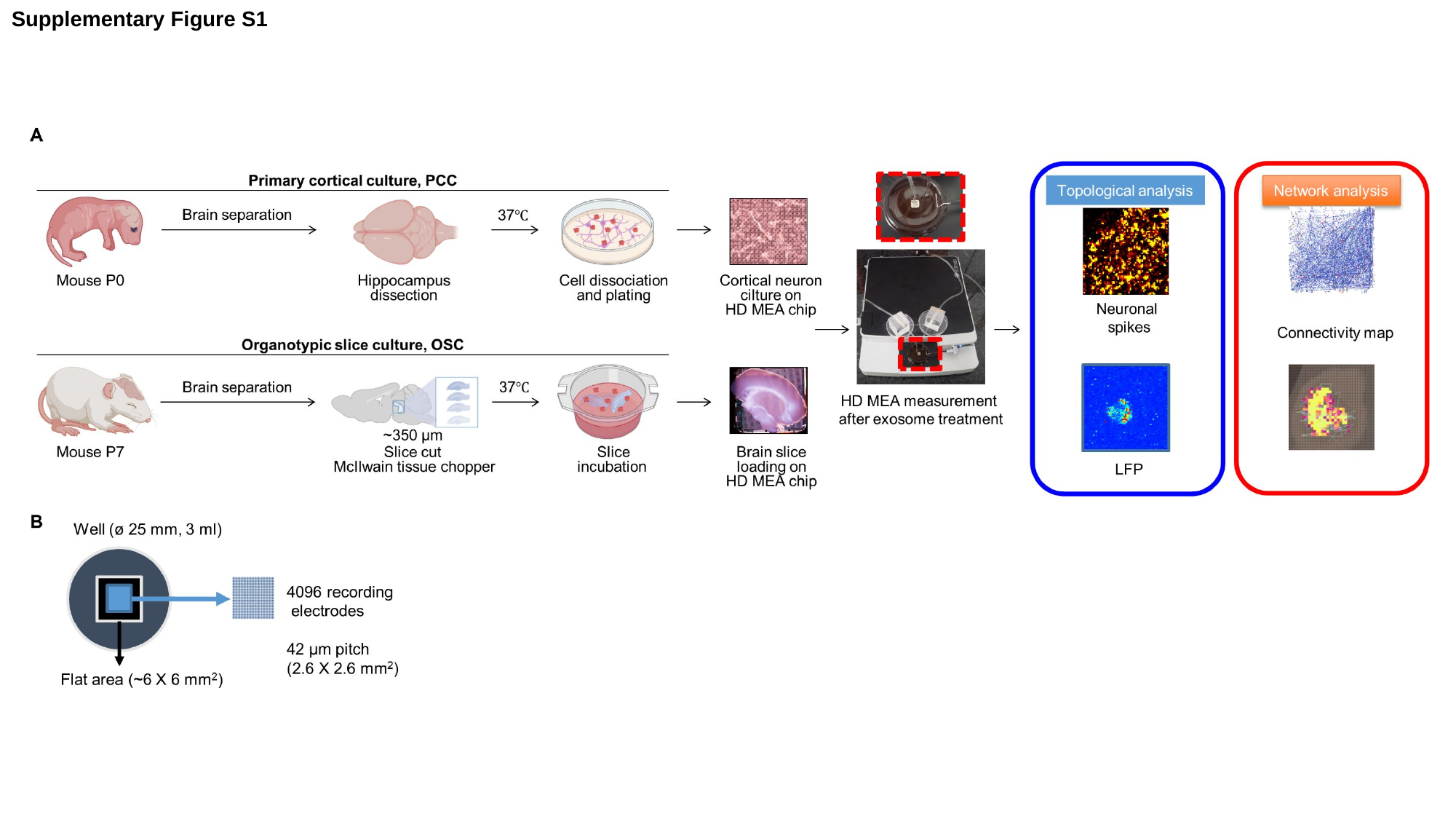

Supplementary Figure S1

## Slide 2
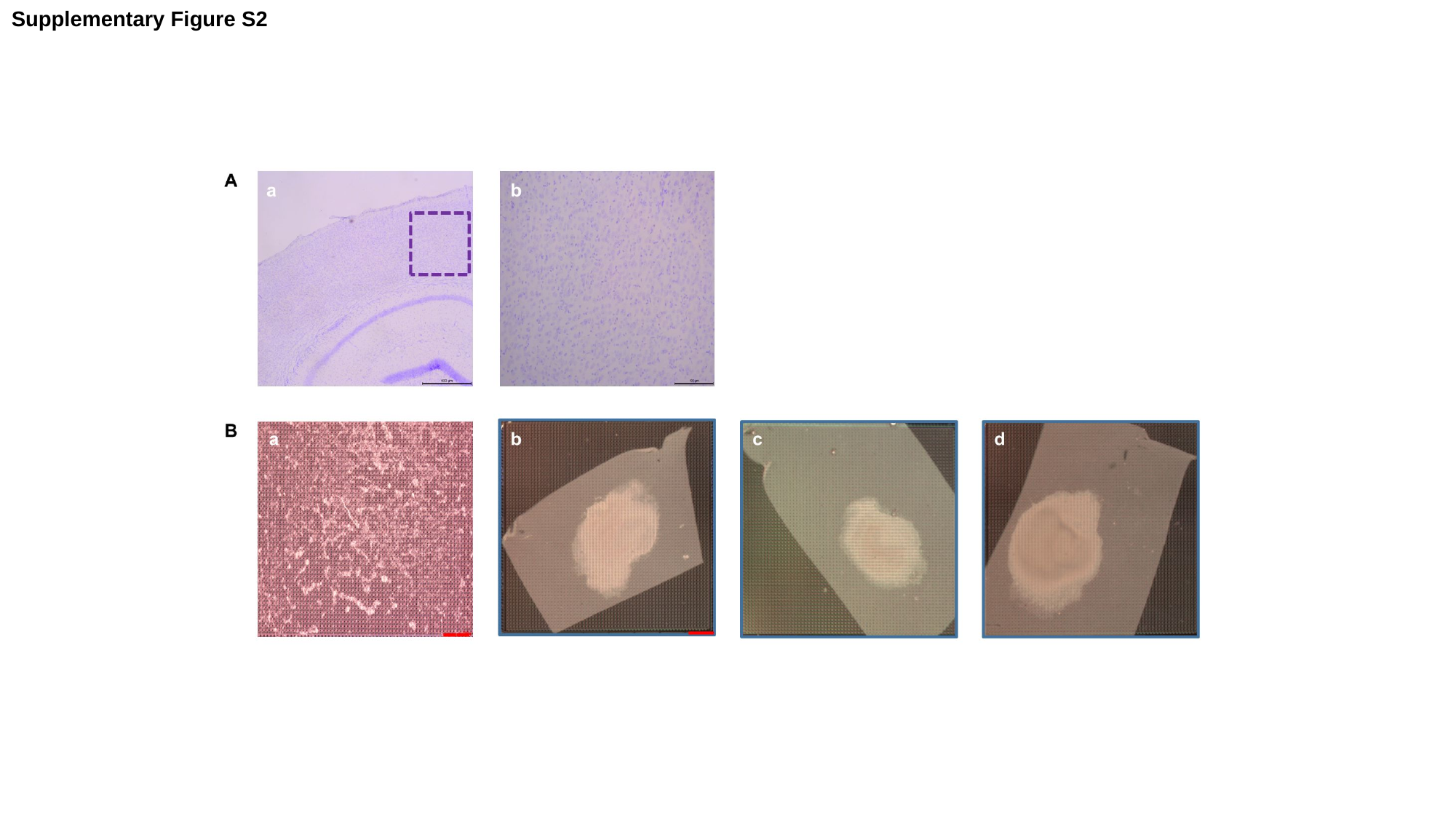

Supplementary Figure S2

## Slide 3
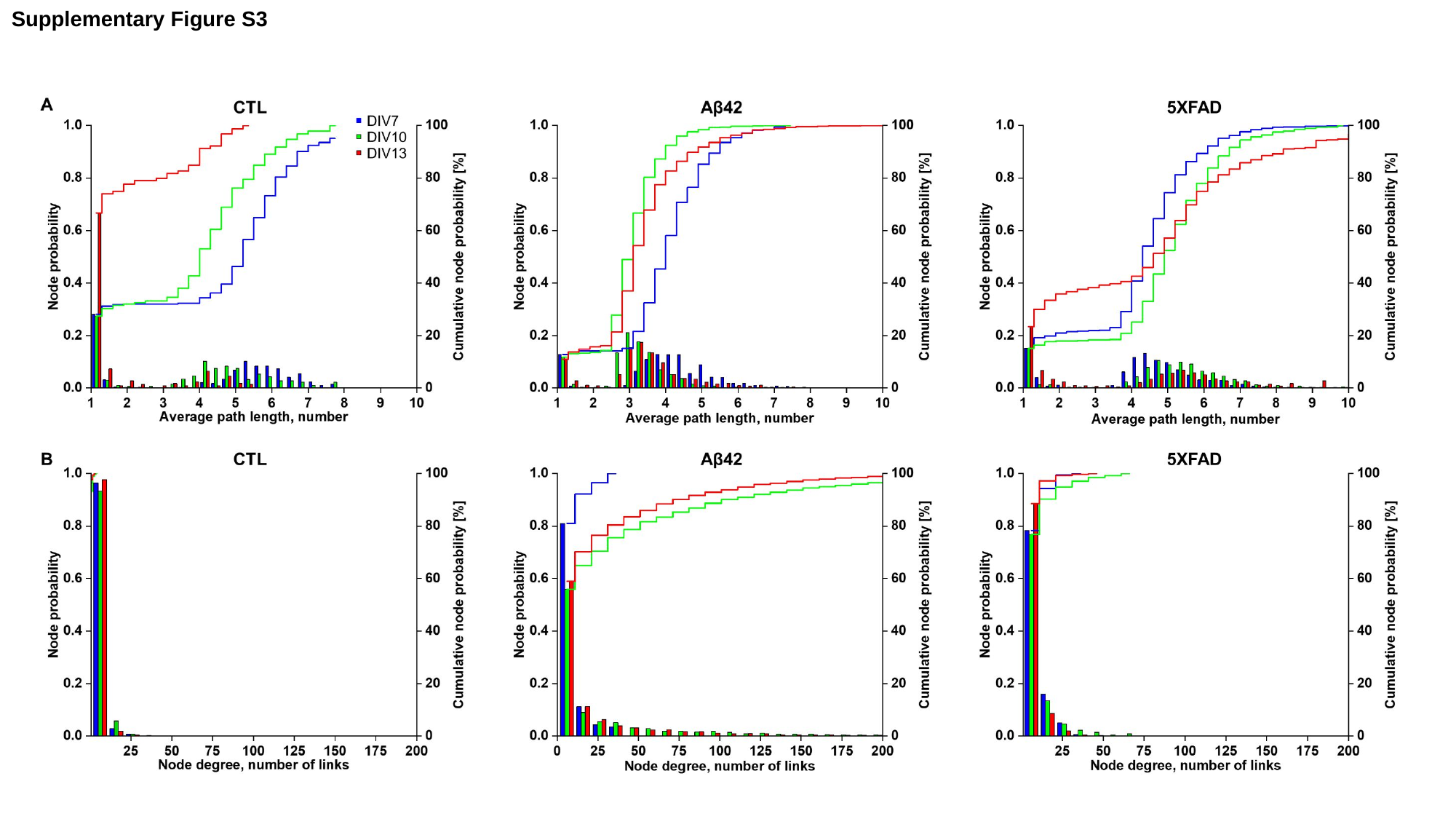

Supplementary Figure S3

## Slide 4
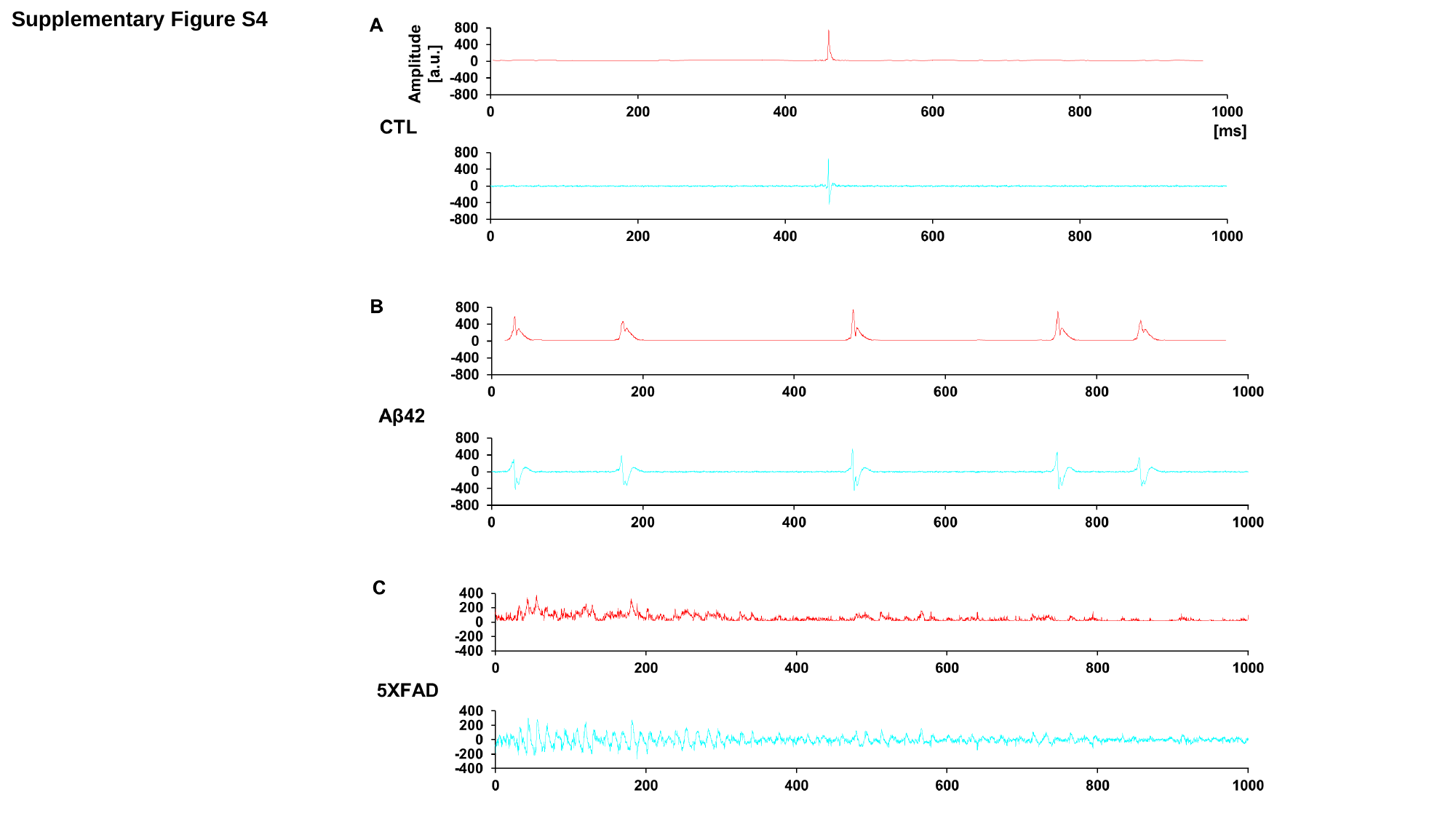

Supplementary Figure S4

## Slide 5
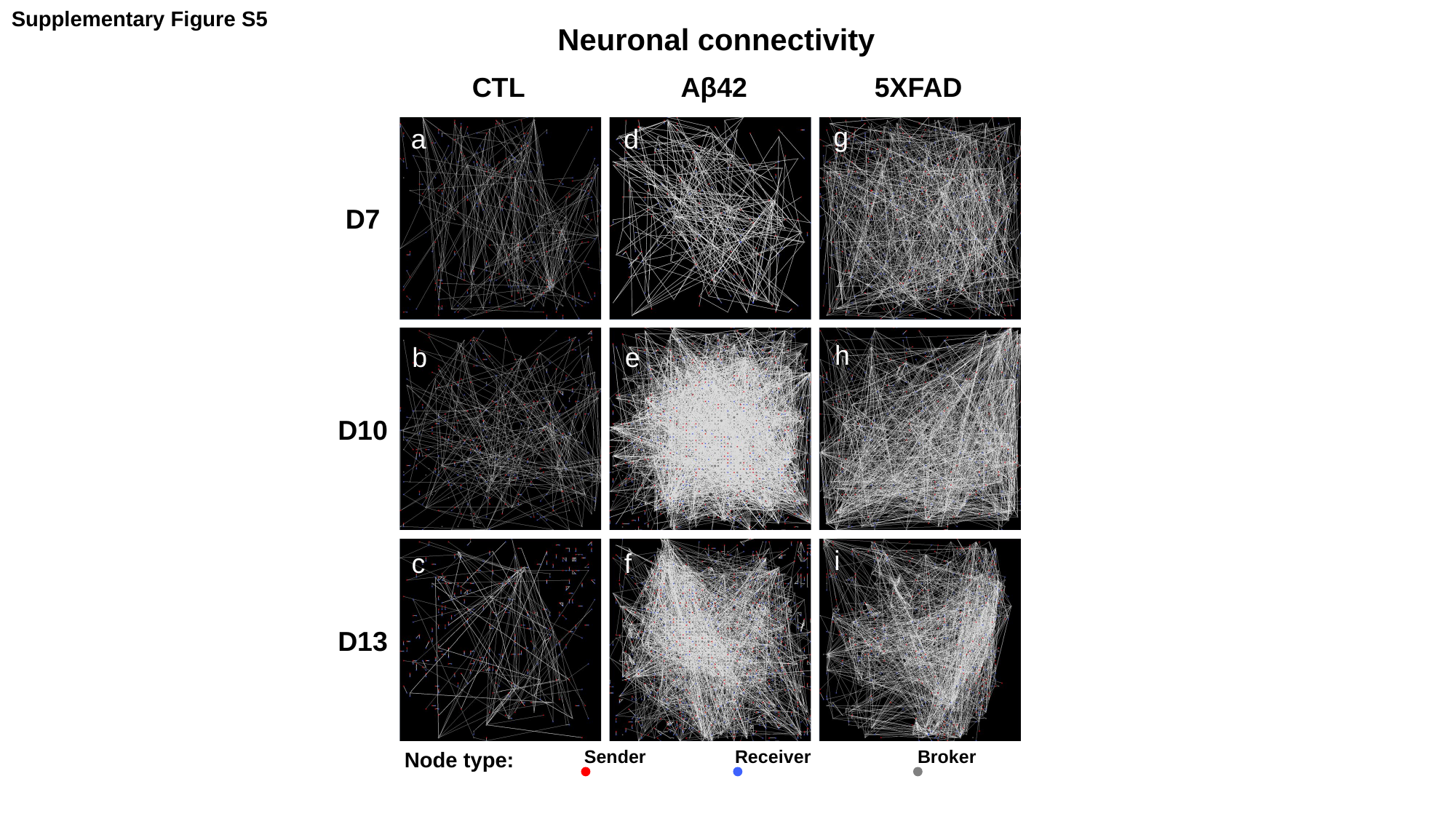

Supplementary Figure S5
Neuronal connectivity
CTL
Aβ42
5XFAD
D7
D10
D13
Sender
Receiver
Broker
Node type:
g
h
i
a
b
c
d
e
f
